# Quality Evaluation and Mathematical Modelling Approach to Estimate the Growth Parameters of Total Viable Count in Sausages with Different Casings

**DOI:** 10.3390/foods11050634

**Published:** 2022-02-22

**Authors:** Chao-Hui Feng

**Affiliations:** 1 School of Regional Innovation and Social Design Engineering, Faculty of Engineering, Kitami Institute of Technology, 165 Koen-cho, Kitami 090-8507, Japan; feng.chaohui@mail.kitami-it.ac.jp; Tel.: +81-0157-26-9390; 2 RIKEN Centre for Advanced Photonics, RIKEN, 519-1399 Aramaki-Aoba, Aoba-ku, Sendai 980-0845, Japan

**Keywords:** modified hog casing, *Baranyi* model, DMfit, modelling, natural hog casing, natural sheep casing

## Abstract

The growth kinetics for the total viable count (TVC) in sausages with modified hog casings (treated by surfactant solutions and slush salt with lactic acid), natural hog casings and sheep casings as a function of the storage time (up to 50 days) were studied for the first time. The growth of TVC was fitted by the *Baranyi* model, and the maximum specific growth rate, lag time and initial and final cell populations were estimated via DMFit. The coefficient of determination of the *Baranyi* model reached 0.94, 0.77 and 0.86 for sausages stuffed in modified hog casings (MHC), control hog casings (CHC) and natural sheep casings (NSC), respectively. The experimental data for the initial populations were 4.69 ± 0.10 log cfu/g for MHC, 4.79 ± 0.10 log cfu/g for CHC and 3.74 ± 0.14 log cfu/g for NSC, whilst the predicted initial cell populations for MHC, CHC and NSC were 4.81 ± 0.20 log cfu/g, 5.19 ± 0.53 log cfu/g and 3.74 ± 0.54 log cfu/g, respectively. Their shelf lives can also be predicted. The results show that the average pH value of MHC samples (6.96 ± 0.01) was significantly lower than that of CHC (7.09 ± 0.01) and NSC (7.05 ± 0.02) samples at day 50 (*p* < 0.05). Sausages with CHC possessed a significant higher water holding capacity (99.48 ± 0.14%) at d 29 than those with MHC (97.40 ± 0.46%) and NSC (98.55 ± 0.17%) (*p* < 0.05). On the last day, the average moisture content for samples with NSC (38.30 ± 3.23%) was significantly higher than that for those with MHC (29.38 ± 2.52%) and CHC (29.15 ± 1.16%) (*p* < 0.05).

## 1. Introduction

Sausages are an important gastronomic and nutritional heritage, which play an important role in people’s daily life [1,2]. Natural sheep and hog casings are extensively utilised in sausage manufacturing due to their special bite and unique flavour. However, the burst incidence generated during sausage stuffing or post-processing frustrates rapid, efficient and large-scale sausage production by sausage manufactures. The necessity to improve the casing properties is increasing as low burst incidences are requested during handling and processing. Consequently, Feng et al. [3] studied the effects of different concentrations of soy lecithin and soy oil associated with slush salt with lactic acid. The results revealed that the casing became more porous after the treatments, and even fat embedded in the casing after immersion vacuum cooling [3]. The interior pressure formed during sausage stuffing can be released and thus can lead to a reduction in the burst incidence, finally increasing the production efficiency. Previous studies regarding the volatile composition changes [4], physicochemical properties [5,6] and microbial attributes [7,8] of sausages using this modified casing have been reported. The prediction of the growth parameters of sausages using a modified casing has not been intensively exploited. As the casing’s microstructure has been changed to be more porous, microorganism invasion can easily occur due to this porous structure. Understanding the growth parameters of microbial attributes can provide useful information on how microorganisms respond to different types of casings. This allows for the sausage industry to comply with regulatory performance standards and to ensure the microbiological safety of sausages using this typical casing.

Compared to time-consuming traditional microbial enumeration methods, predictive mathematical modelling is gaining increasing interest among academic researchers and food manufactures [9,10,11]. The Gompertz model has been used to estimate the growth parameters of raw chicken meat with different types of spice extracts under different storage temperatures [12]. The results showed that the lag phase duration, maximum population and maximal growth rate can be sufficiently estimated with a coefficient of determination (R^2^) range from 0.93 to 0.99 [12]. The cross-contamination of *Escherichia coli O157:H7* on the surface of ready-to-eat meat products during slicing has been modelled by generic and exponential models [13]. The results revealed that a low surface transfer count with <5 log CFU may lead to a higher probability of cross-contamination. The model developed by the authors may also apply to the prediction of slicing cross-contamination with a lower detectable level (e.g., <4 log CFU) [13]. The growth parameters of lactic acid bacteria and the total viable count in vacuum-packaged Irish cooked sausages with different cooling methods were calculated by the *Baranyi* model [2]. The results showed that the *Baranyi* model (with no lag) can accurately describe the growth of lactic acid bacteria in sausages cooled by commercial cooling and immersion vacuum cooling, with an R^2^ of 97.4% and 98.3%, respectively. All these studies accentuate an unyielding interest in the application of predictive modelling as a powerful and promising method that can predict microbial parameters, especially when the experimental data are difficult to collect or insufficient to obtain.

The main objective of this study was to (a) monitor the microbiological (total viable count, TVC) and physicochemical (pH, water holding capacity, moisture content) characteristics of uncooked sausages stuffed in different casings and (b) evaluate their quality in terms of microbial dynamics using mathematical modelling.

## 2. Materials and Methods

### 2.1. Casing Modifications and Sausage Preparation 

Natural hog casings (Pakumogu.com, Niigata Prefecture, Japan) were modified with a surfactant solution and slush salt with lactic acid (19.50 mL/kg). The specific modification procedures can be found in Feng et al. (2019) [5]. Before sausage stuffing, all the modified solution and slush salt in modified casings were totally removed by rinsing with distilled water for 10 min. Lean pork (visible fat removal, 56.85%) and pork back fat (24.36%) were sterilely cut into small pieces. All the lean pork, pork back and aforementioned ingredients were mixed well using Chinese white wine (ethanol content: 52%, *v*/*v*), cured for 1 h in the fridge (4 °C) and stuffed by a stuffing machine (STX-4000-TB2-PD-BL, Electric Meat Grinder & Sausage Stuffer, STX international, Lincoln, NE, USA) using natural sheep, hog and modified casings. The sausages were sectioned by twisting and dried in a sterilised oven at 45 °C for 24 h, followed by ageing for another 48 h at 20 °C. The detailed processing can be found in Feng and Makino (2020) [6] and Feng et al. [14]. The sausage cuts were vacuum packaged, stored at 4 °C and analysed on days 1, 7, 16, 22, 29, 36, 43 and 50 for physicochemical analysis. 

### 2.2. Physicochemical Analysis 

The water holding capacities (WHC) of sausages with different casings were evaluated by a modified centrifuge method [15]. The meat cores without casings were wrapped in a dry cheesecloth and centrifuged for 10 min at 4 °C at the speed of 9000× *g*. The WHC of the sample was calculated as follows:

(1) $$\mathrm{WHC}\;(\%)=\frac{M_{1}}{M_{2}}\times \;100\%$$

where *M_2_* and *M_1_* are the weights before and after centrifugation.

As for pH analysis, 10 g of sample was aseptically homogenised with 90 mL distilled water for 1 min and measured using a digital pH meter (Module-C, Sevenexcellence Instrument, Greifensee, Switzerland). This method was in accordance with Zdolec et al. (2008) [16]. 

Regarding moisture content, 5 g minced sausage samples were dried in an oven (105 ± 1 °C) until constant weight. The sausage weights before and after drying were calculated as the moisture content. 

### 2.3. Bacterial Enumeration and Modelling the Growth Parameters 

A slice of 10 g sausage with a casing was sterilely weighted and homogenised with 90 mL of 0.85% saline for 2 min using a laboratory stomacher (E-Mix Primo, ASONE interscience Co., Inc., Osaka, Japan). Samples were tenfold serial diluted using 0.85% saline. An amount of 0.1 mL of diluted samples was spread onto CicaMedia Standard plate count agar (Kanto Chemical Co., Inc., Tokyo, Japan, pH: 7.0 ± 0.2) for the enumeration of total viable counts (TVCs) and incubated at 37 °C for 48 h. 

The *Baranyi* model [17] was utilised to fit the microbial data using DMFit (Institute of Food Research, Norwich, UK, http://www.ComBase.cc, accessed on: 28 January 2022) in ComBase. 

The *Baranyi* model is shown as follows:


(2) $$\ln(N(t))=\ln(N_{0})+{µ}_{\max}A(t)-\ln\;\left[1+\frac{e^{{µ}_{\max}A\left(t\right)-1}}{e^{\left(N_{\max}-N_{0}\right)}}\right]$$

(3) $$A(t)=t+\frac{1}{{µ}_{\max}}\;\ln\;(e^{{µ}_{\max^{\prime }}}+e^{-{µ}_{\max}\lambda }-e^{-{µ}_{\max}\left(t+\lambda \right)\;})$$

where ln (N(*t*)), ln (N_0_) and ln (N_max_) are the log of the cell population at time *t* (d (day)) (CFU/g), the log of the initial cell population (CFU/g) and the log of the final cell population, respectively; µ_max_ and *λ* are the maximum specific growth rate (day^−1^) and lag time (day), respectively. 

All the experiments were implemented in triplicate. MHC samples were analysed on days 0, 7, 16, 22, 29, 36, 43 and 50, while CHC and NSC samples were analysed on days 0, 2, 7, 16, 29, 36, 43 and 50. 

### 2.4. Statistical and Accuracy Analysis 

The effects of different casings on WHC, pH, moisture content and TVC were analysed by a one-way ANOVA programme (Statistics 28, IBM, Armonk, NY, USA). 

The coefficient of determination (R^2^) was used for estimating the goodness of fit of the model. It is commonly believed that an R^2^ value over 0.91 is an excellent prediction, whilst an R^2^ value between 0.66 and 0.81 is recognised as an acceptable level for quantitative prediction [18]. The range of the R^2^ value between 0.82 and 0.90 is reported to be a good prediction [19].

## 3. Results and Discussion

### 3.1. Effects of Different Casings on Quality Attributes of Sausages during Long-Term Storage

#### 3.1.1. pH

As an essential parameter, pH can greatly affect the colour, water holding capacity, protein properties, flavour and shelf life of meat products [20]. The pH values for samples stuffed in natural sheep casings were significantly higher than those with other casings, irrespective of the storage times (*p* < 0.05) (Table 1). The increasing pH may be due to nitrogen compounds produced by proteolysis [20]. It is well known that natural casings come from the submucose layer of animal small intestines, and the thickness of sheep and hog submucose layers is, on average, 0.11 mm and 0.32 mm, respectively [21]. It can be deduced that the chemical activities will be higher in the thin casing (sheep casing) than other casings as the casing acts as a barrier for preventing the sausage batters from contacting with the exterior environment. This barrier ability will be weaker with the thinner casing (i.e., sheep casings). Marapana et al. also found that higher cooking loss occurred in the sheep casing compared with the hog casing [22]. The authors attributed this to the greater thickness of the hog casing which prevented the loss of inside materials [22]. At day 50, it can be found that the average pH value of the sausages stuffed in modified hog casings (6.96 ± 0.01) was significantly lower than that of control hog casings (7.09 ± 0.01) and natural sheep casings (7.05 ± 0.02) (*p* < 0.05). The porous structure of the modified casings may explain this observation. It has been reported that casings after modification become more porous [3], leading to lipid peroxidation and the growth of lactic acid bacteria. With regard to the different storage days, the pH value of sausages stuffed in the modified hog casing (6.96 ± 0.01) at day 22 was significantly higher than that at day 1 (6.90 ± 0.01) (*p* < 0.05). A similar observation was noted for the control hog casing (6.98 ± 0.01 at day 22 vs. 6.93 ± 0.02 at day 1; *p* < 0.05) and natural sheep casings (7.01 ± 0.01 at day 22 vs. 6.97 ± 0.01 at day 1; *p* < 0.05). 

#### 3.1.2. Water Holding Capacity

Water holding capacity is a parameter used to evaluate the ability of meat to hold water during handling, processing and storage [23], which is one of the most important properties to assess sausage quality [24]. As shown in Table 1, sausages with control hog casings possessed a significant higher water holding capacity (99.48 ± 0.14%) at day 29 than those with the modified hog casing (97.40 ± 0.46%) and natural sheep casing (98.55 ± 0.17%) (*p* < 0.05). There are several factors that will influence the water holding capacity of the meat: meat condition before and after slaughter, cooking, chilling [25], protein, salt concentration [26], moisture content, etc. Zayas (1997) stated that protein interactions with water were the most important functional property which affects the retention of water against gravity [27]. Ma et al. (2018) indicated that the increase in NaCl could enhance the WHC value due to the chloride ions penetrating into myofilaments, leading to swelling and an ion “cloud” around the filaments produced [26]. Concerning the current study, it may be due to the higher moisture content. It can also be observed that a significantly higher moisture content was achieved for the control hog casings (34.39 ± 3.57%) in comparison with the modified hog casing (27.76 ± 2.30%) (*p* < 0.05). On the last storage day (i.e., day 50), the WHC value of sausages stuffed in hog casings (98.22 ± 0.12%) was significantly lower than that of those using sheep casings (98.85 ± 0.12%) (*p* < 0.05). Marapana et al. (2018) also found that the WHC value of sausages stuffed in sheep casings (44.21 ± 0.20) was significantly higher than that of those in hog casings (43.32 ± 0.14) after 3 months of storage (*p* < 0.05) [22]. 

#### 3.1.3. Moisture Content

For evaluating sausage quality, moisture is also an important attribute and related to sensory and textural characteristics [28]. It is essential to control the moisture content to avoid undesirable microorganism growth such as fungi and mould [29]. There were no significant differences with regard to the different storage days for samples using modified hog casings (*p* > 0.05), indicating the moisture content stability. As for sausages with control hog casings, the moisture content of sausages at day 50 (29.15 ± 1.16%) was significantly lower than that at day 1 (39.63 ± 3.46%) (*p* < 0.05). Regarding samples with natural sheep casings, the moisture content of sausages at day 1 (27.39 ± 2.21%) was lower than that at day 50 (38.30 ± 3.23%) at a significance of *p* < 0.05. The mean moisture content of CHC samples (39.63 ± 3.46%) was significantly higher than that of NSC samples (27.39 ± 2.21%) (*p* < 0.05) at day 1, while at day 50, NSC samples’ moisture content (38.30 ± 3.23%) was significantly higher than that of CHC samples (29.15 ± 1.16%) (*p* < 0.05). The water produced by microbial metabolism may explain this phenomenon. 

### 3.2. Effects of Different Casings on Microbial Attributes of Sausages during Long-Term Storage

#### 3.2.1. Growth Parameters of Total Viable Count

Table 2 displays the calculated maximum specific growth rates and the initial and final cell populations for the total viable count of sausages using different casings. As an important indicator to evaluate the shelf life of the products, the total viable count specifies the quantity of the dominant microorganism. There was no lag time for all samples irrespective of the casings used. The experimental data for the initial populations were 4.69 ± 0.10 log cfu/g for MHC, 4.79 ± 0.10 log cfu/g for CHC and 3.74 ± 0.14 log cfu/g for NSC, whilst the predicted initial cell populations for MHC, CHC and NSC were 4.81 ± 0.20 log cfu/g, 5.19 ± 0.53 log cfu/g and 3.74± 0.54 log cfu/g, respectively. The average maximum specific growth rates for samples with modified hog, control hog and natural sheep casings were 0.13 ± 0.03, 0.20 ± 0.09 and 1.31 ± 0.40 day^−1^, respectively. Table 3 shows that the cell population of MHC samples at day 16 (6.71 ± 0.56 log cfu/g) was significantly higher than that at day 0 (4.69 ± 0.10 log cfu/g), whilst for NSC samples, the cell populations achieved significantly higher values at day 2 (6.35 ± 0.35 log cfu/g) than at day 0 (3.74 ± 0.14 logcfu/g) (*p* < 0.05). These results imply that microorganisms in the sausages stuffed in sheep casings grew faster than those in the other casings. This may be due to the comparably thinner property for sheep casings than for hog and modified hog casings. The average thickness of NSC (0.019 ± 0.003 mm) was significantly thinner than that of CHC (0.030 ± 0.010 mm) and MHC (0.170 ± 0.050 mm) (*p* < 0.05) [30]. The thicker modified hog casing (almost nine times thicker than the sheep casing) worked as a thick “wall” to hinder the invasion of the bacteria, which can be attributed to the comparable lower maximum specific growth rate of sausages using the modified hog casings. It can also be found that the cell population of MHC samples at day 50 (7.17 ± 0.19 log cfu/g) was significantly lower than that of sausages stuffed using CHC (8.64 ± 0.29 log cfu/g) (*p* < 0.05). Marapana et al. (2018) also observed significant differences in the total plate count regarding the different types of casings with the storage period [22]. Although their sausage treatments (freezing cooked sausages) were quite different from the current study (aged uncooked sausages), the total plate count of sausages with hog casings increased gradually after the 5th week (i.e., 35 days), and sausages with sheep casings achieved the highest value (5.1 × 10^6^ cfu/g: 6.71 log cfu/g) in the 5th week. Settanni et al. (2020) studied the microbiological and physicochemical composition of salamis made from four different meats (beef, pork, horse and wild boar) during ripening with the condition of 90% humidity and 13 °C for up to 45 day [31]. *Lb. sakei* was reported to be dominant in all salamis irrespective of the meat types, which could be attributed to its high competitiveness, psychrotrophic profile and salt tolerance [31]. The effects of different cooling methods and casings (natural and modified hog casings) on TVC and lactic acid bacteria (LAB) were investigated [8]. According to principal component analysis, TVC lay close to LAB, implying that LAB may be the main bacteria for the TVC. This is consistent with the observation of Settanni et al.’s study [31]. The dominant species in the current study will be identified in a future study. 

#### 3.2.2. Prediction of Shelf Life

**Figure 1:**
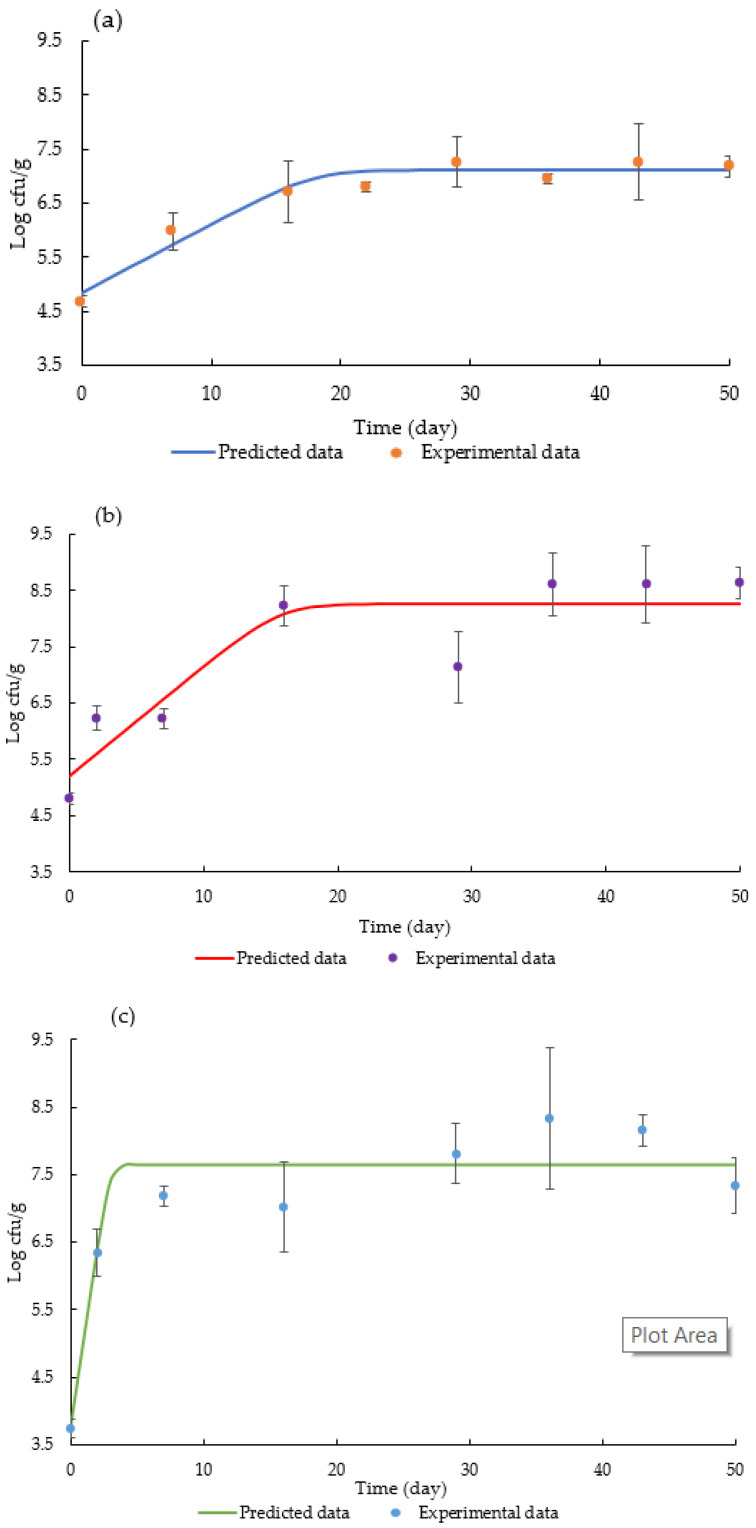
Fitting of the *Baranyi* model to the population of total viable counts of sausages stuffed in: (**a**) modified hog casings (MHC); (**b**) control hog casings (CHC); and (**c**) natural sheep casings (NSC).

As illustrated in Figure 1, the fitted data can sufficiently describe the growth tendency of the sausages with different casings. The R^2^ value of samples stuffed in modified hog casings, control hog casings and natural sheep casings using the *Baranyi* model reached 0.94, 0.77 and 0.86, respectively. The standard error of fit was 0.22, 0.69 and 0.54, respectively (Table 2). In recent decades, the *Baranyi* model has been intensively studied [32,33,34,35], and it has been proved to have the best goodness of fit for numerous microorganisms’ growth curves [33,36]. If the model can accurately predict the data, it will not only reduce the tedious experimental work but also reveal how the microbial behaviour responds to the different treatments (i.e., different casings). Moreover, it can be a useful tool for forecasting the shelf life of food products. The value of 7 log cfu/g is commonly regarded as the safety limit for meat products [37,38]. In this way, the shelf lives of these uncooked sausages with modified hog casings, control hog casings and natural sheep casings were predicted to be 19, 10 and 3 days, respectively. Modified sausage casings presented a longer shelf life than sausages using other casings.

The current study found the following:

1. The growth parameters of sausages with different types of casings, especially the modified casing, were estimated by using the *Baranyi* model for the first time, and the coefficient of determination for sausages stuffed in modified casings reached 0.94. These results can be useful to understand how the microorganism behaviour acts under different types of sausage casings.
2. The quality attributes of sausages stuffed in modified casings, control hog casings and natural sheep casings as a function of the long-term storage time have been clearly elucidated. The data obtained from the current study can provide useful information for sausage manufacturing in regard to producing fresh sausages using different sausage casings.

## 4. Conclusions

The pH, water holding capacity and moisture content of uncooked sausages stuffed in modified hog casings, control hog casings and natural sheep casings were investigated in this study. A structured kinetic model was applied to describe the microbial growth during 50 days of cold anaerobic storage for sausages stuffed using different casings. The *Baranyi* model can sufficiently describe the total viable count in sausages with modified hog casings. The maximum specific growth rate of TVC in sausages with NSC was revealed to be the highest, which may be due to the thinner casing property. This study could provide useful information for evaluating the shelf life of sausages with different types of casings.

## Figures and Tables

**Table 1:** Chemical property comparison for sausages with different casings during 50 days of cold storage (4 °C).

Parameters	Samples	Day 1	Day 7	Day 16	Day 22	Day 29	Day 36	Day 43	Day 50
pH	MHC	6.90 ± 0.01 ^Aa^	6.88 ± 0.01 ^Aa^	6.88 ± 0.01 ^Aa^	6.96 ± 0.01 ^Ba^	6.95 ± 0.02 ^Ba^	7.00 ± 0.01 ^Cb^	7.00 ± 0.02 ^Ca^	6.96 ± 0.01 ^Ba^
	CHC	6.93 ± 0.02 ^BEa^	6.92 ± 0.01 ^BEb^	6.93 ± 0.00 ^BEb^	6.98 ± 0.01 ^CDa^	6.90 ± 0.01 ^Eb^	6.97 ± 0.01 ^Da^	7.05 ± 0.01 ^Ab^	7.09 ± 0.01 ^Fb^
	NSC	6.97 ± 0.01 ^EFb^	6.96 ± 0.01 ^Fc^	6.97 ± 0.01 ^BEFc^	7.01 ± 0.01 ^DGb^	7.04 ± 0.01 ^DCc^	7.00 ± 0.01 ^EGb^	7.10 ± 0.01 ^Ac^	7.05 ± 0.02 ^Cc^
WHC (%)	MHC	96.26 ± 1.20 ^Aa^	99.01 ± 0.08 ^BCa^	98.78 ± 0.14 ^BDa^	97.70 ± 0.29 ^BDa^	97.40 ± 0.46 ^ADa^	97.87 ± 0.29 ^BDb^	97.64 ± 0.41 ^ACDb^	98.58 ± 0.20 ^BDab^
	CHC	99.01 ± 0.13 ^Bb^	98.96 ± 0.68 ^BCa^	99.09 ± 0.10 ^Ba^	99.02 ± 0.22 ^Ba^	99.48 ± 0.14 ^Bb^	99.50 ± 0.12 ^Ba^	97.21 ± 0.06 ^Ab^	98.22 ± 0.12 ^Ca^
	NSC	98.19 ± 0.51 ^Ab^	98.65 ± 0.36 ^Aa^	98.79 ± 0.22 ^Aa^	94.55± 7.16 ^Aa^	98.55 ± 0.17 ^Ac^	98.35 ± 0.30 ^Ab^	98.88 ± 0.13 ^Aa^	98.85 ± 0.12 ^Ab^
MC (%)	MHC	29.49 ± 2.56 ^Aa^	32.70 ± 0.91 ^Aa^	29.35 ± 0.95 ^Aa^	29.76 ± 1.08 ^Aa^	27.76 ± 2.30 ^Aa^	27.34 ± 3.27 ^Ab^	31.91 ± 2.79 ^Ab^	29.38 ± 2.52 ^Ab^
	CHC	39.63 ± 3.46 ^ACb^	34.88 ± 3.47 ^BCa^	38.24 ± 1.69 ^ACb^	34.55 ± 1.27 ^BCa^	34.39 ± 3.57 ^BCb^	43.50 ± 0.49 ^Aa^	33.22 ± 2.12 ^BCb^	29.15 ± 1.16 ^Bb^
	NSC	27.39 ± 2.21 ^BDa^	30.61 ± 0.12 ^BCDa^	33.48 ± 0.32 ^CEFc^	34.41 ± 2.92 ^CEFa^	31.59 ± 1.02 ^BDFab^	27.60 ± 1.32 ^Db^	44.03 ± 2.09 ^Aa^	38.30 ± 3.23 ^Ea^

Note: Sausages stuffed in MHC: modified hog casing; CHC: control hog casing; NSC: natural sheep casing; WHC: water holding capacity; MC: moisture content. Different lower letters within a column indicate a significant difference (*p* < 0.05). Different upper letters within a row indicate a significant difference (*p* < 0.05).

**Table 2:** Predicted shelf life and calculated model parameters for modelling the total viable count growth of sausages with different casings.

Parameters Estimated from *Baranyi* Model	MHC	CHC	NSC
Initial cell population (ln (N_0_), log cfu/g)	4.81 ± 0.20	5.19 ± 0.53	3.74 ± 0.54
Maximum specific growth rate (μ_max_, day^−1^)	0.13 ± 0.03	0.20 ± 0.09	1.31 ± 0.40
Lag time (λ, day)	--	--	--
Final cell population (ln (N_max_), log cfu/g)	7.09 ± 0.10	8.26 ± 0.34	7.64 ± 0.22
Coefficient of determination (R^2^)	0.94	0.77	0.86
Standard error (SE) of fit	0.22	0.69	0.54
Predicted shelf life (day)	19	10	3

Note: Sausages stuffed in MHC: modified hog casing; CHC: control hog casing; NSC: natural sheep casing.

**Table 3:** Total viable count comparison for sausages with different casings during 50 days of cold storage (4 °C).

	Day 0	Day 2	Day 7	Day 16	Day 22	Day 29	Day 36	Day 43	Day 50
MHC (log cfu/g)	4.69 ± 0.10 ^Aa^	---	5.97 ±0.34 ^ABa^	6.71 ± 0.56 ^Ba^	6.80 ± 0.10 ^B^	7.26 ± 0.47 ^Ba^	6.95 ± 0.10 ^Ba^	7.25 ± 0.70 ^Ba^	7.17 ± 0.19 ^Ba^
CHC (log cfu/g)	4.79 ± 0.10 ^Aa^	6.24 ± 0.22 ^Ba^	6.22 ± 0.18 ^Ba^	8.22 ± 0.36 ^CDb^	---	7.13 ± 0.64 ^BCa^	8.60 ± 0.56 ^Db^	8.62 ± 0.69 ^Da^	8.64 ± 0.29 ^Db^
NSC (log cfu/g)	3.74 ± 0.14 ^Aa^	6.35 ± 0.35 ^Ba^	7.18 ± 0.15 ^BCDa^	7.02 ± 0.66 ^BCab^	---	7.81 ± 0.44 ^BCDa^	8.34 ± 1.05 ^Db^	8.16 ± 0.23 ^CDa^	7.34 ± 0.42 ^BDab^

Note: Sausages stuffed in MHC: modified hog casing; CHC: control hog casing; NSC: natural sheep casing. Different lower letters within a column indicate a significant difference (*p* < 0.05). Different upper letters within a row indicate a significant difference (*p* < 0.05). “---” means “no available data”.

## Data Availability

Data are contained within this article.
